# Proteomic Profiling and Artificial Intelligence for Hepatocellular Carcinoma Translational Medicine

**DOI:** 10.3390/biomedicines9020159

**Published:** 2021-02-06

**Authors:** Nurbubu T. Moldogazieva, Innokenty M. Mokhosoev, Sergey P. Zavadskiy, Alexander A. Terentiev

**Affiliations:** 1 Laboratory of Bioinformatics, Institute of Translational Medicine and Biotechnology, I.M. Sechenov First Moscow State Medical University (Sechenov University), 119991 Moscow, Russia; 2 Department of Biochemistry and Molecular Biology, N.I. Pirogov Russian National Research Medical University, 117997 Moscow, Russia; imokhosoev@mail.ru (I.M.M.); terentjev_aa@rsmu.ru (A.A.T.); 3 Department of Pharmacology, A.P. Nelyubin Institute of Pharmacy, I.M. Sechenov First Moscow State Medical University (Sechenov University), 119991 Moscow, Russia; zavadskiy_s_p@staff.sechenov.ru

**Keywords:** proteomics, artificial intelligence, biomarkers, hepatocellular carcinoma, translational medicine

## Abstract

Hepatocellular carcinoma (HCC) is the most common primary cancer of the liver with high morbidity and mortality rates worldwide. Since 1963, when alpha-fetoprotein (AFP) was discovered as a first HCC serum biomarker, several other protein biomarkers have been identified and introduced into clinical practice. However, insufficient specificity and sensitivity of these biomarkers dictate the necessity of novel biomarker discovery. Remarkable advancements in integrated multiomics technologies for the identification of gene expression and protein or metabolite distribution patterns can facilitate rising to this challenge. Current multiomics technologies lead to the accumulation of a huge amount of data, which requires clustering and finding correlations between various datasets and developing predictive models for data filtering, pre-processing, and reducing dimensionality. Artificial intelligence (AI) technologies have an enormous potential to overcome accelerated data growth, complexity, and heterogeneity within and across data sources. Our review focuses on the recent progress in integrative proteomic profiling strategies and their usage in combination with machine learning and deep learning technologies for the discovery of novel biomarker candidates for HCC early diagnosis and prognosis. We discuss conventional and promising proteomic biomarkers of HCC such as AFP, lens culinaris agglutinin (LCA)-reactive L3 glycoform of AFP (AFP-L3), des-gamma-carboxyprothrombin (DCP), osteopontin (OPN), glypican-3 (GPC3), dickkopf-1 (DKK1), midkine (MDK), and squamous cell carcinoma antigen (SCCA) and highlight their functional significance including the involvement in cell signaling such as Wnt/β-catenin, PI3K/Akt, integrin αvβ3/NF-κB/HIF-1α, JAK/STAT3 and MAPK/ERK-mediated pathways dysregulated in HCC. We show that currently available computational platforms for big data analysis and AI technologies can both enhance proteomic profiling and improve imaging techniques to enhance the translational application of proteomics data into precision medicine.

## 1. Introduction

Hepatocellular carcinoma (HCC) is a multifactorial heterogeneous disease and the most common primary malignant tumor of the liver with increasing incidence rate worldwide [1]. HCC is the fifth diagnosed cancer and the second most frequent cause of cancer-related deaths in men and the ninth cancer case and the sixth cause of deaths from cancers in women [2]. Liver cirrhosis is a main cause of HCC and together with inflammation associated with hepatitis B virus (HBV) or hepatitis C virus (HCV) accompanies early stages of HCC [3,4]. Consequently, diagnostic and prognostic biomarkers with high specificity and sensitivity for HCC diagnosis at an early stage and differentiation between HCC and non-HCC diseases are of crucial importance. Moreover, monitoring patient’s postoperative status and treatment efficacy along with evaluation of disease progression and metastasis risk to predict cancer recurrence are needed [5].

HCC is typically diagnosed by liver biopsy or cross-sectional liver imaging techniques such as contrast-enhanced computer tomography (CT) and magnetic resonance imaging (MRI) [6]. These techniques are useful for tumor staging and detecting extrahepatic metastases, which involve, mostly, lungs, lymph nodes, bone, adrenal glands, and peritoneum [7]. Usage imaging criteria according to Liver Imaging Reporting and Data System (LI-RADS) and introduction of novel imaging technologies such as contrast-enhanced liver ultrasound can improve early diagnosis and differentiating HCC from non-HCC liver diseases to increase surveillance of HCC patients [8,9]. However, some limitations in imaging approaches such as time consuming and low sensitivity dictate necessity of developing both novel screening methods and highly sensitive and specific biomarkers for HCC early diagnosis and prognosis.

Currently available integrative genomic/epigenomic/transcriptomic/proteomic profiling approaches and biomarker assay techniques provide multifaceted insight into biomarker discovery. Comprehensive multiomics profiling enables differentiating early and advanced HCCs as well as HCC from chronic liver diseases, even without knowledge of the clinical symptoms [10]. Additionally, this allows assessing intra-tumoral phenotypic heterogeneity and uncovering individual variability and alterations in unique gene expression patterns, which underlie tumor initiation and progression [11]. Multiomics-based platforms are used for molecular classification of HCC subtypes characterized by different driver genes to provide deeper insight into cancer pathogenesis and to evaluate the potency of genomic, epigenomic, and proteomic signatures as HCC biomarkers [12].

The challenge in multiomics technologies is the accumulation of a huge amount of very heterogeneous raw data stored in different data formats. A large amount of complex heterogeneous data is referred to as big data, which are described by top “V’s” characteristics such as value, volume, velocity, variety, veracity, and variability [13]. Big data analytics in cancer implies the integration, analysis, interpretation, validation, and quality control of large datasets from thousands of patients. This requires suitable and promising open-source distributed data processing software platforms.

Significant progress has been achieved due to the application of artificial intelligence (AI) technologies, which enhance healthcare data collection and interpretation. This is provided by computer-based algorithms for data analysis and by the construction of predictive models for improving image recognition and representation in HCC diagnosis and prognosis [14]. Additionally, AI arises as a powerful tool in the analysis and integration of complex and heterogeneous datasets obtained due to multiomics profiling for disease staging, prediction of disease recurrence, monitoring treatment response, and the identification of diagnostic, prognostic, and predictive biomarkers [15].

Proteomics is a large-scale investigation and analysis of proteins aimed to identify and characterizing proteomes as a complete protein composition of a cell or tissue [16]. Proteomics implies protein distribution profiling and protein expression/activity patterning and protein-protein interaction identification. Current integrated proteomic profiling technologies use hybrid platforms based on multi-dimensional (MD) separations and three-dimensional (3D) liquid chromatography (LC) and have provided powerful solutions [17]. Currently available proteomic data from MS-based proteomic experiments are integrated in public repositories such as ProteomeXchange Consortium (http://www.proteomexchange.org, accessed on 28 December 2020) [18], Proteomics Identification (PRIDE) (http://www.ebi.ac.uk/pride, accessed on 28 December 2020) [19], Human Plasma Peptide Atlas (http://www.peptideatlas.org, accessed on 28 December 2020) [20]. These repositories provide efficient and reliable dissemination, comparative analysis, interpretation, and extraction of proteomic data.

Translational medicine implies, on the one hand, application of new knowledge into clinical practice to increase efficacy of a disease diagnosis and therapeutic strategies and, on the other hand, to facilitate generation of new hypotheses from clinical observations. The aim of translational medicine is combination of benchside, bedside and community in the enhancement of patient’s care decision making [21]. Integrated data from diverse omics technologies enable the large-scale identification of novel molecular biomarkers for translating them into clinical practice. Thus, translating knowledge on a biomarker structure, functions, and expression into clinical practice should enable HCC early diagnosis, prognosis, and assessment of treatment efficacy. However, limited success has been achieved in translating cancer biomarker proteomic profiling into clinical practice.

Our review focuses on the recent advancements in the integrative proteomic profiling strategies and emerging AI technologies for discovery of novel biomarkers for HCC early diagnosis and prognosis. We discuss proteomic signatures of HCC, starting from conventional and promising biomarkers and alterations in cell signaling pathways involved in hepatocarcinogenesis. This is followed by consideration of the latest findings in exploring novel proteomic biomarker candidates with emphasis on their translational application.

## 2. Proteomic Profiling Technologies and Big Data

**Figure 1:**
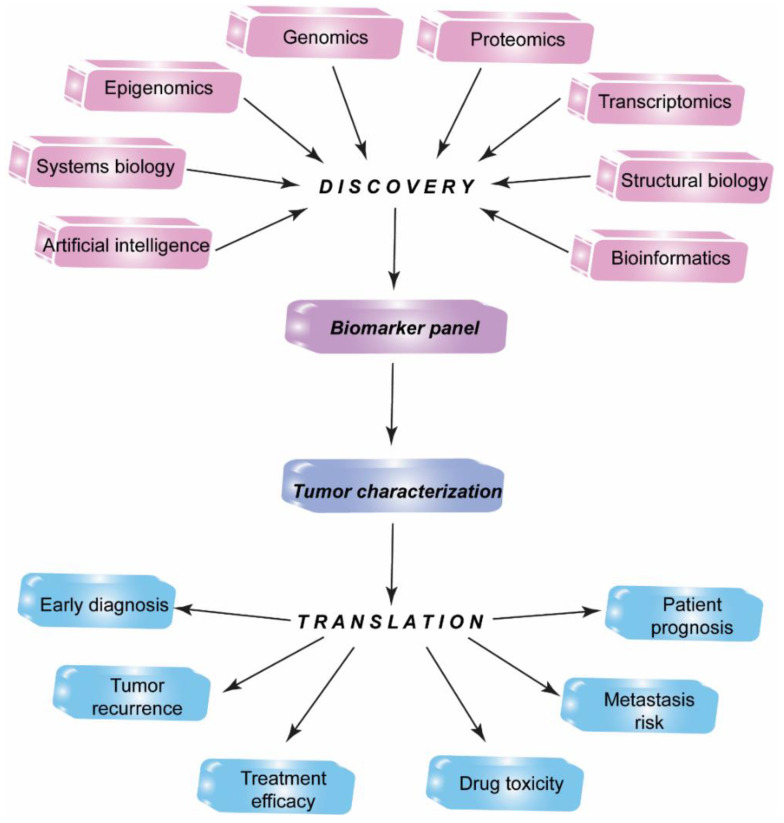
Multiomics biomarker discovery for translational medicine. Integrated high-throughput proteomics, genomics, epigenomics, transcriptomics, bioinformatics approaches along with systems biology, structural biology, artificial intelligence techniques allow identification of a candidate biomarker panel to discover new biomarkers for translation into practical medicine.

The identification, standardization, and validation of effective tumor biomarkers can dramatically influence the cancer diagnosis, prognosis, and anti-cancer drug development. In the past, the focus of cancer biomarker research has been on the usage of a single experimental technique such as immunoprecipitation analysis or ELISA. Currently, biomarker candidates are being identified with the use of integrated technologies including genomics, epigenomics, transcriptomics, proteomics, systems biology, bioinformatics, and molecular imaging approaches (Figure 1) [22].

The process of candidate biomarker identification typically involves analysis of tissue samples and blood serum or plasma to reveal gene expression or epigenome patterns and protein or metabolite distribution profiles. This strategy is based on comparative study of tissue or blood samples to identify genes, proteins, and metabolites changed in patients as compared to those in healthy donors. In this manner, a panel of biomarkers can be constructed with sensitivity and specificity necessary for disease detection and monitoring to be ultimately applied in clinical practice. Simultaneous analysis of a biomarker panel and quantitative verification and validation of candidate biomarkers represent an obligatory and rate-limiting process, which can be enhanced by AI approaches and neural network algorithms for the identification of serological liver marker profiles [23].

Correct sample preparation is a critical step in biomarker profiling. This is especially important if a biomarker candidate presents in a sample at an extremely low concentration (at ng/mL level). Standard multi-step sample preparation can cause protein degradation and sample loss, which lead to result variations. Consequently, sample fractionation, protein enrichment by immuno-affinity depletion, and scalable automated proteomic pipeline are usually employed to decrease the sample complexity and to increase the assay sensitivity, specificity, high-throughput capability, and multiplicity [24]. Integrated proteomic sample preparation technologies for the fast and deep plasma proteome profiling at native pH values such as mixed-mode ion exchange-based method has been developed [25]. Additionally, simple and integrated spintip-based proteomics technology (SISPROT) for sample preparation in combination with spatial proteome profiling using laser capture microdissection (LCM) technique has been proposed to enable precise dissection of specific cells in tumor sample at a single cell resolution. For example, LCM-SISPROT technology based on immunochemistry has been used to identify HCC cell population specific spatial proteome [26]. Moreover, single cell resolution multiomics technologies provide valuable opportunity to measure various biomolecules at their low concentrations [27,28].

Proteomic profiling enables simultaneous analysis of thousands of proteins and identification of hundreds of biomarkers in a single sample and their large-scale characterization, quantification, and validation. Thus, proteomic profiling is a useful tool for the evaluation of changes in unique tumor-specific gene expression patterns, which lead to shifts in a protein amount and distribution in tissues and body fluids. Changes in gene expression signatures and protein distribution patterns have been used to assess tumor stage, tumor recurrence, post-operative outcomes, and anti-cancer treatment response [29]. Moreover, such a strategy has proved to be effective for determining intratumoral heterogeneity and finding correlations between gene expression/protein concentration and disease progression levels.

Currently available proteomic profiling technologies involve 2-dimensional polyacrylamide gel electrophoresis (2-DE) and liquid chromatography (LC) combined with protein and peptide identification and analysis using various tandem mass-spectrometry (MS) types. High-throughput integrated proteomic approaches include liquid chromatography-tandem mass-spectrometry (LC-MS/MS), liquid chromatography-selected reaction monitoring mass spectrometry (LC-SRM MS), matrix-assisted laser-desorption ionization time of flight (MALDI-TOF) or surface-enhanced laser desorption/ionization time of flight (SELDI-TOF) MS as well as protein chip and microarray technologies [30,31,32]. For example, a combination of 2-DE and LC-MS has been used in targeted proteomics to discover biomarkers for early HCC diagnosis and distinguishing high-risk chronic hepatitis C virus infected patients from HCC patients [33]. Subsequent quantitative verification and validation of candidate biomarkers was performed by SRM-MS.

**Figure 2:**
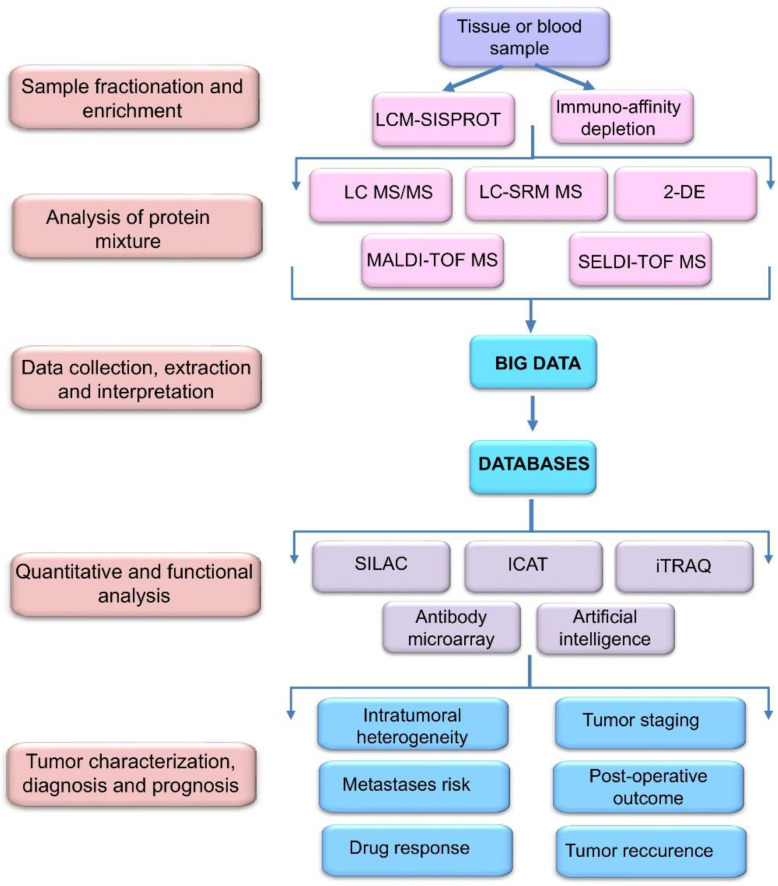
Workflow of biomarker identification, quantification, validation, and verification using proteomic profiling technologies. Tissue and blood samples are first fractionated and enriched using laser capture microdissection spintip-based proteomics (LCM-SISPROT) and immuno-affinity depletion. Then, biomarker candidates are identified using two-dimensional gel electrophoresis (2-DE) and liquid chromatography (LC) in combination with various types of mass-spectrometry (MS) including selected reaction monitoring (SRM), matrix-assisted laser-desorption ionization time of flight (MALDI-TOF) and surface-enhanced laser desorption/ionization time of flight (SELDI-TOF) MS. Large-scale integrated proteomic profiling leads to the accumulation of a huge amount of data (big data), which are collected and stored in special databases for further processing using stable isotope labeling by amino acids in cell culture (SILAC), isotope coded affinity tag (ICAT), and isobaric tags for relative and absolute quantitation (iTRAQ) and other technologies.

Capability of current MS/MS and LC-MS methods has been greatly enhanced by the advancements in quantitative proteomic analysis by mass spectrometry using non-radioactive isotope labeling to determine differences in the abundance of proteins and peptides from different samples/treatments in a single experiment. These include stable isotope labeling by amino acids in cell culture (SILAC), isotope coded affinity tag (ICAT), and isobaric tags for relative and absolute quantitation (iTRAQ) technologies [34]. These approaches allow studying quantitative changes in a whole proteome and gene expression level as well as functions of a certain protein biomarker (Figure 2).

Liquid biopsies in combination with LC-MS/MS-based proteomics arise as a powerful high-sensitive and non-invasive platform for the identification of biomarkers at extremely low concentrations in a complex mixture [35]. Unlike conventional tissue biopsy, liquid biopsy is a non-invasive approach, which allows repeated analysis to enable monitoring tumor progression, metastasis, and recurrence, as well as treatment response. This enables identification and accounting novel biomarker candidates circulating in the bloodstream with sensitivity, specificity, positive, and negative predictive values reaching up to 100% [36].

The latest achievements in big multiomics data have led to the creation of various platforms for extracting, summarizing, and interpreting knowledge for their translating into precision medicine [37]. Collection and representation of proteomic data can be made in public repositories such as Global Proteome Machine (GPM) database created for proteomic information obtained from tandem MS (https://www.thegpm.org/, accessed on 28 December 2020) [38] and The Peptide Atlas SRM Experiment Library (PASSEL) created for storage of data obtained in SRM experiments and supported by Institute of Systems Biology, Seattle, WA, USA (http://www.peptideatlas.org/passel, accessed on 28 December 2020) [39].

Current multiomics (genomics, epigenomics, transcriptomics, metabolomics, and proteomics) technologies lead to the accumulation of a huge amount of data, which requires clustering and finding correlations between various datasets and developing predictive models with the use of bioinformatics, image analysis, and computational data mining methods [40]. Big data characteristics are: a huge amount of data (volume), speed of their collection, processing and analysis (velocity), dataset complexity and heterogeneity (variety), quality and reliability as well as predictive value of data (veracity), data consistency over the time (variability), and utility to patients and clinicians (value). These characteristics require reducing data dimensionality, filtering and pre-processing, which can be achieved due to emerging AI technologies.

## 3. Artificial Intelligence in HCC Imaging and Biomarker Exploring

AI is a promising approach to overcome accelerated data growth, complexity, and heterogeneity within and across data sources. AI provides an automated integration of multiomics data and prediction of whole-organism level phenotype from molecular-level genotype through the identification of driver mutations in genes and changes in protein expression, which underlie disease initiation and progression [41].

Machine learning (ML) is a fundamental concept of AI, which uses computer algorithms to learn from an experience and to build models for prediction or decision making with the use of sample data known as training data. Several studies have been carried out exploiting ML technologies for uncovering biomarker signatures, which allow assessment of cancer outcomes and recurrence. For example, a recent study used The Cancer Genome Atlas (TCGA), AMC and Inserm databases, and ML algorithms to identify gene signatures that could predict early HCC recurrence [42]. The constructed ML-based model showed 74.19% accuracy of the prediction, and the selected mutant genes were verified. Additionally, probe electrospray ionization (PESI) MS in combination with AI have been employed to assess the overall diagnostic accuracy of two algorithms, support vector machine (SVM) and random forest (RF), in HCC detection [43]. This approach showed bench-top size, minimal sample preparation, and short working time as well as high accuracy, specificity, and sensitivity in HCC diagnosis. The overall diagnostic accuracy exceeded 94% for the both AI algorithms.

**Figure 3:**
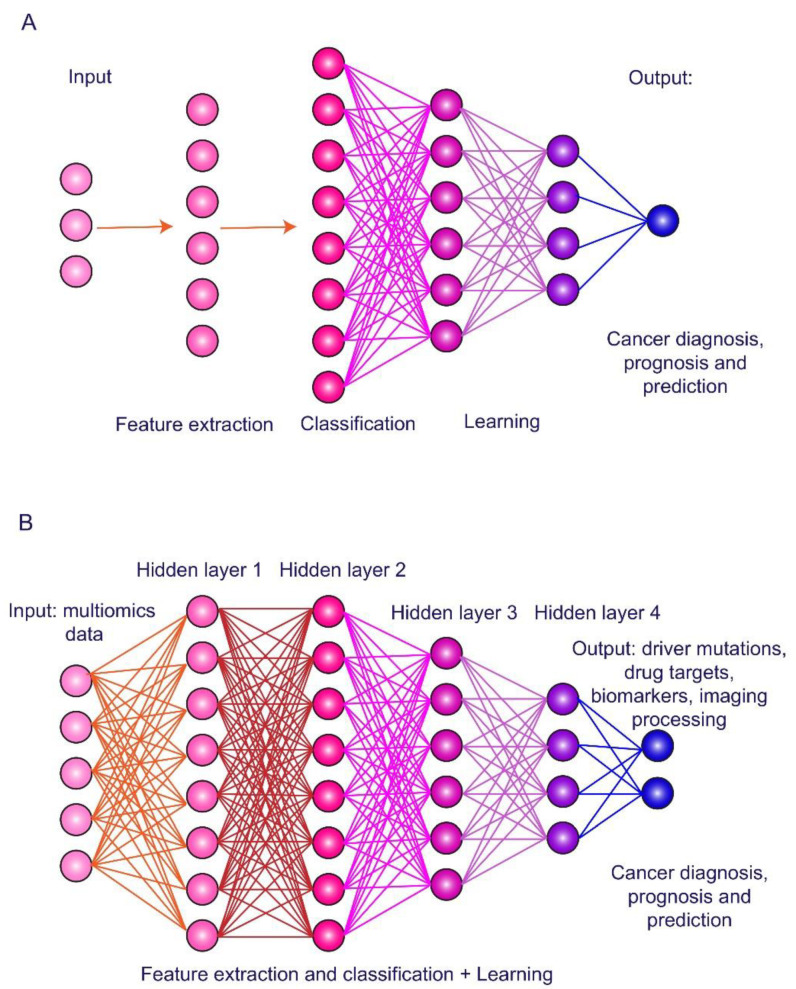
Principles of artificial intelligence technologies. (**A**) Machine learning is usage of computer algorithms to learn from an experience and to build predictive models based on training data. (**B**) Deep learning is data representation learning through artificial neuronal networks and multiple layers for feature extraction and classification to object recognition and decision making. Multiomics profiling and imaging data can be used as inputs, while identification of driver mutations, drug targets, plasma biomarkers, and image processing are outputs for hepatocellular carcinoma diagnosis, prognosis, and prediction.

A part of ML methods is deep learning (DL) based on artificial neuronal networks (ANNs), which have layered structure with interconnected nodes and an activation function among them (Figure 3). DL refers to data representation learning with multiple levels of abstraction through multiple processing layers in the network to construct computational models for object recognition. ANNs are trained using back-propagation algorithm and utilizing test samples to improve data representation. DL algorithms allow discovering intricate structure in large datasets to indicate changes in internal parameters of a machine that are used to compute the representation in each layer from the representation in the previous layer [44]. DL has been proposed as a tool for improving feature extraction from raw data and classification to increase performance for high-level feature representation [45].

In recent years, DL algorithms and models have been, mostly, applied to cancer image-based diagnosis, prognosis, and prediction [46,47]. For example, convolutional neural networks (CNNs) have allowed interpretation of HCC images in the identification of liver masses and recognizing specific features of pathological lesions [48]. Another example is joint multiple fully connected CNNs, which have shown superior performance in HCC nuclei grading [49]. Additionally, DL models combined with imaging techniques helped in differentiating HCC patients who can benefit from interventional treatment. Indeed, trained and validated residual CNN models in combination with CT imaging showed high performance for prediction of post-operative HCC patients’ response to trans-arterial chemoembolization (TACE) [50]. Furthermore, visualization of 3D CNN analysis and DL-based radiomics strategy, which utilized quantitative analysis of pre-operative contrast-enhanced ultrasound (CEUS) cines have enabled accurate prediction of HCC patients’ response to TACE [51].

Combination of DL models with three-phase contrast-enhanced CT showed accuracy, which was similar to four-phase CT in differentiating HCC from focal liver lesions [52]. The achieved performance was 83.3%, 81.1%, and 85.6% accuracy for models A, B, and C suggesting that multiphase CT protocol can be optimized by removal of pre-contrast phase to reduce radiation dose. ML-based radiomics combined with quantitative imaging features extracted from triphasic CT scans can enhance HCC diagnosis in cirrhotic patients with indeterminate liver nodules [53]. Additionally, multiphase CT radiomics in combination with deep DL models have been shown to improve prediction accuracy in HCC early recurrence after surgically removed tumor [54].

In recent years, some progress has been achieved in the application of DL algorithms, not only to the image-based cancer detection and treatment prediction, but also to the integration of multiomics data. This allows novel biomarker discovery through the identification of driver mutations and dysregulated signaling pathways for tumor molecular classification and drug response prediction. For example, 360 HCC patients’ data have been exploited to construct DL-based survival-sensitive model using sequencing of RNA (RNA-Seq), miRNA (miRNA-Seq), and methylation data from TCGA [55]. This model allowed patient classification into two optimal subtypes significantly differed by survival rate. A more aggressive subtype was associated with frequent inactivation mutations in the *TP53* gene, along with overexpression of stemness markers (*KRT19* and *EPCAM*) and tumor marker *BIRC5*, and activated Wnt and PKB/Akt signaling pathways. Additionally, DL algorithms based on conventional regression approach have been used to construct predictive model of HCC recurrence after liver transplantation in 563 patients. This multi-center study showed that tumor diameter, age, and levels of protein biomarkers such as alpha-fetoprotein (AFP) and prothrombin induced by vitamin K absence or antagonist-II (PIVKA-II) are the largest weighted parameters in the AI-based Model of Recurrence after Liver Transplantation (MoRAL-AI) [56].

Additionally, evaluation of serum AFP level and albumin-bilirubin (ALBI) grade, along with liver cirrhosis, tumor margin, and radiomics signatures have increased ML-based contrast-enhanced CT performance accuracy in the prediction of HCC recurrence rate after curative tumor resection [57]. Furthermore, DL algorithms based on multiomics data from TCGA database were used for exploring prognostic indicators for 320 HCC patients [58]. Genetic alterations such as the *FAT3* and *RYR2* mutations were identified in addition to sinusoidal capillarization, prominent nucleoli and karyotheca, the nucleus/cytoplasm ratio, and infiltrating inflammatory cells as the main underlying features of tumor risk score in HCC.

All the above-mentioned examples illustrate that AI algorithms can enhance both imaging techniques and multiomics data-based large-scale biomarker identification, quantification, and validation for HCC diagnosis and prognosis. However, there is still limited success in the implementation of AI technologies in uncovering genotype-phenotype relationships in cancer. The reasons are high heterogeneity of multiomics (genomics, epigenomics, transcriptomics, metabolomics, and proteomics) data and insufficiency of available datasets to accurately train models.

## 4. Conventional Biomarkers of Hepatocellular Carcinoma

Currently, recommended biomarkers for combined testing for HCC includes AFP, Lens culinaris agglutinin (LCA)-reactive L3 glycoform of AFP (AFP-L3), and des-gamma-carboxyprothrombin (DCP). In addition to HCC diagnosis, they are useful as predictive biomarkers for monitoring the tumor recurrence and treatment responsiveness. Among them, AFP remains a primary molecular biomarker for HCC diagnosis and prognosis recognized as a “golden standard” among serum tumor markers [59,60].

### 4.1. Alpha-Fetoprotein and Its Glycoform

In 1956, Swedish researchers Bergstrand and Czar, using paper electrophoresis, found a new human protein fraction in the fetal blood serum with the mobility of alpha-globulins [61]. Later, in 1963, Russian scientists Garry Abelev and Yuri Tatarinov reported on immunochemical discovery of a new antigen specific for chemically induced mouse hepatoma and human primary liver cancer, respectively [62,63]. Afterwards, this oncofetal antigen was designated as alpha-fetoprotein and has become recognized as the first embryo-specific and cancer-associated biomarker (for more details, see history of AFP discovery in [64]).

**Figure 4:**
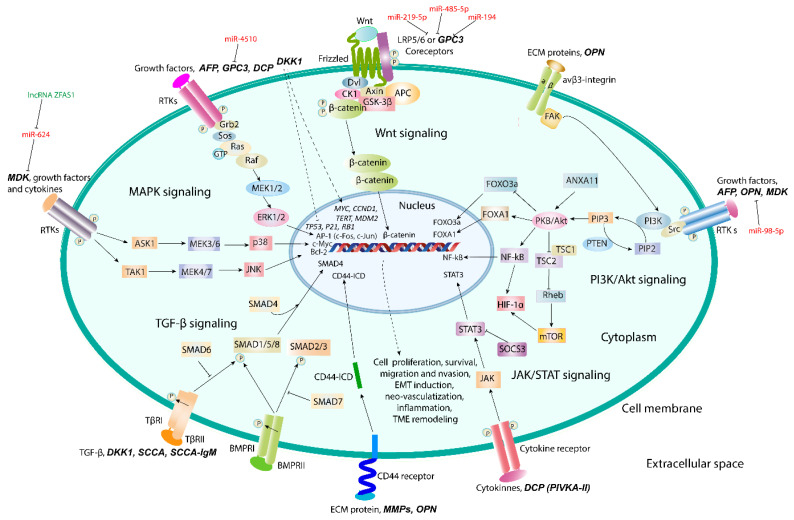
Cell signaling pathways involved in hepatocellular carcinoma progression. Proteomic biomarkers act through MAPK, Wnt/β-catenin, PI3K-Akt, JAK/STAT, and TGF-β/SMAD signaling pathways for cell proliferation, survival, migration and invasion, induction of epithelial-to-mesenchymal transition, angio- and vasculogenesis, inflammation, and tumor microenvironment remodeling. Regulation of a biomarker expression by non-coding RNAs and changes in gene expression signatures are shown.

AFP has an ability of dual regulation of cell proliferation and survival depending on cell type and AFP concentration, along with immunosuppressive activity and capability of binding and transportation of different hydrophobic ligands [65,66,67,68]. It has been shown that structure/function relationship exists between growth factors, cell adhesion proteins, and AFP, and that the effects of AFP may be provided through the mitogen-activated protein kinase (MAPK)-signaling pathway [69,70,71]. Additionally, an ability of cytoplasmic AFP to co-localize and interact with caspase-3 and to block TNF-alpha-related apoptosis-inducing ligand (TRAIL) and all-trans retinoic acid (ATRA)-mediated apoptosis has been observed [72]. Cytoplasmic AFP can also function as a regulator of phosphatidyl-inositol-3-kinase (PI3K)/Akt signaling in human hepatoma cell lines [73]. Mir-1236 miRNAs cause inhibition of the PI3K/Akt-mediated pathway and down-regulate AFP expression followed by the inhibition of AFP-stimulated cell proliferation, migration, invasion, and vasculogenic mimicry [74]. This was accompanied by phosphatase and tensin homolog (PTEN) accumulation and the inhibition of malignant phenotype of hepatoma cells. AFP co-localized and interacted with PTEN inducing, thereby, CXCR4 chemokine receptor expression by activating Akt/mTOR signaling pathway and stimulating migration of hepatoma cells (Figure 4) [75].

AFP is the only biomarker which has passed through all five phases of biomarker identification and validation procedure [76]. AFP is widely used in clinics as an independent factor for HCC late stage, early recurrence, and poor prognosis [77,78]. Serum concentration of AFP alone or in combination with ultrasound showed good accuracy in HCC diagnosis, and sensitivity and specificity of the test with a threshold of AFP at 400 ng/mL were better than those at a threshold of 200 ng/mL [79]. Additionally, usage of standard deviation of AFP and rate of AFP elevation as well as patient-specific risk factors such as age, platelet count, and smoking status has been reported to improve prognostic accuracy of the test as compared to usage of only AFP level [80]. Level of AFP ˃400 µg/mL index tumor size ˃5 cm and vascular invasion have been shown to strongly associate with extrahepatic metastases in HCC, especially when combined with into multi-parametric metastasis prediction criterion [81].

However, because of low sensitivity and specificity, diagnostic value of the test for AFP is not high and utility for HCC surveillance is controversial. Sensitivity and specificity of AFP have been reported to vary from 39% to 64% and from 76% to 91%, respectively [82]. Besides, approximately 40% of early HCC patients and 15–20% of advanced HCC patients have been shown to be AFP-negative, i.e., serum AFP level is less than 20 ng/mL [83]. Nevertheless, test for AFP improves performance of diagnosis and served as a valuable surveillance test for HCC associated with HCV-caused cirrhosis with normal level of alanine aminotransferase (ALT) [84].

AFP-L3 is a glycoprotein, which contains α-1,6-fucose attached to *N*-acetylglucosamine at its reducing terminus, while AFP-L3 aberrant fucosylation is used in identifying HCV, chronic hepatitis B (CHB), and liver cirrhosis (LC) patients with high risk of HCC development. Based on data of aberrant protein fucosylation during HCC development, analysis of relationships between fucosylation index, tumor genesis, and progression in HBV-associated HCC was performed for blood serum proteomic profiling using MALDI-TOF mass spectrometry [85]. When combined with serum AFP detection (AFP > 20 ng/mL), the sensitivity/specificity of aberrant fucosylation test for HCC improved to 78/88%, 85/88%, and 89/91% in all serum samples, HBV-associated chronic liver diseases and HBV-associated cirrhosis, respectively.

A meta-analysis of fifteen studies with 4465 patients showed that high pre-treatment level of AFP-L3 implies poor overall survival (OS) and poor prognosis in HCC patients with low AFP level [86]. Additionally, an increase in the percentage of AFP-L3 over the total AFP level (>10%) is highly specific for small-sized HCC. However, since serum levels of these proteins are independent of each other, combined measurement of the two or three biomarkers can increase their sensitivity and accuracy for HCC diagnosis. High or increasing serum AFP and AFP-L3 levels have been shown to be indicative of large tumor size, advanced stage, and extra-hepatic metastasis in HCC [87].

### 4.2. Des-Gamma-Carboxyprothrombin

DCP is also known as PIVKA-II and an abnormal prothrombin without carboxylation of γ-carbon atom in several glutamic acid residues in γ-carboxyglutamic (Gla) domain located at its *N*-terminal region. Consequently, DCP does not have coagulation activity [88]. Instead, it exhibits growth factor activity and directly stimulates DNA synthesis and HCC cell proliferation in both autocrine and paracrine manner. This is achieved through Janus kinase-1 (JAK1)-signal transducer and activator of transcription-3 (STAT3) signaling pathway by binding to c-Met cell surface receptor. Additionally, DCP can promote vascular endothelial cell proliferation and migration through MAPK-mediated signaling pathway [89] as confirmed by immunohistochemical analysis, which reveals the existence of correlation between DCP expression and HCC tumor size and hyper-vascularization [90]. In cultured HCC cells, DCP stimulates HCC growth and metastasis through activation of MMP2 and MMP-9 due to binding to c-Met receptor and causing its phosphorylation followed by epidermal growth factor receptor (EGFR) activation with subsequent ERK1/2, MEK1/2, and c-Raf (MAPK signaling) stimulation [91].

Serum level of DCP has been reported to correlate with HCC aggressiveness and poor prognosis [92]. Elevated level of DCP in HCC correlates with deficiency in carboxylation of coagulation factors at their γ-glutamyl residues leading to prolonged activated partial prothrombin time (APPT). This can be achieved due to down-regulation of vitamin K epoxide reductase complex subunit 1 (VKORC1) through stimulation of p-ERK and suppression of mTOR signaling [93].

Currently, DCP is considered as a phase II biomarker, which is more specific than total AFP level in detecting HCC and more reliable than AFP as a prognostic tool for HCC recurrence and patient survival after hepatectomy, liver transplantation, radio-frequency ablation, and TACE treatment [94]. Additionally, in randomized trials for hepatitis C antiviral long-term treatment against cirrhosis, DCP has shown sensitivity and specificity comparable to those of AFP [95].

Simultaneous assessment of gender, age, AFP, AFP-L3, and DCP, a panel denoted as GALAD score, in the same serum samples of 685 HCC patients showed that 55.8% were AFP-positive, 34.1% were AFP-L3-positive, and 54.2% were DCP-positive and the number of biomarkers present clearly reflected the extent of HCC and patient outcomes decreasing after treatment [96]. Moreover, simultaneous multi-center measurement of AFP, AFP-L3, and PIVKA-II in newly diagnosed HCC patients showed that AFP has the best diagnostic performance as a single biomarker for HCC. However, diagnostic value of AFP has improved when combined with PIVKA-II but adding AFP-L3 did not enable distinguishing between HCC and non-HCC liver cirrhosis [97]. Nevertheless, another study showed that combined testing for all three biomarkers improves diagnostic accuracy as compared to each biomarker alone [98]. The sensitivity and specificity of the combination of three biomarkers were 87.0% and 60.1%, respectively, in total HCC cases, and 75.7% and 60.1%, respectively, in early HCC cases.

Additionally, DCP concentration in the blood serum can increase in patients with vitamin K deficiency and patients who have obstructive jaundice. In these conditions, DCP designated as NX-DCP has an increased amount of Gla residues. NX-DCP is differed from DCP itself by the expression level and biological properties. Since NX-DCP can be produced in HCC tissues, it has been proposed as a useful biomarker for clinical evaluation of the tumor severity and duration of survival among HCC patients [99]. The reason for DCP and NX-DCP increase may be hypoxia caused by tumor growth that impairs vitamin K uptake to induce DCP expression. Higher NX-DCP expression is associated with significantly low histological grade and portal vein invasion than lower NX-DCP level [100]. Additionally, DCP-positive (≥40 mAU/L), NX-DCP-positive ((≥90 mAU/L), and DCP/NX-DCP ratio ≥1.5 cases have been shown to closely relate to malignant properties of HCC.

## 5. Promising Proteomic Biomarkers of HCC

Promising proteomic biomarkers such as glypican-3 (GPC3), osteopontin (OPN), midkine (MDK), dickkopf-1 (DKK-1), alpha-l-fucosidase, squamous cell carcinoma antigen-1 (SCCA-1), Golgi protein-73 (GOLPH2), carcinoembryonic antigen (CEA), vascular endothelial growth factor (VEGF), and matrix metalloproteinases-2 and -9 (MMP-2 and MMP-9) along with genomic driver mutations, miRNAs, lncRNAs, circulating tumor DNA (ctDNA) and circulating exosomes are presently being extensively studied for HCC diagnosis and prognosis, and treatment monitoring [101,102,103,104,105,106,107,108,109]. However, most of the newly discovered biomarkers are still complementary to AFP since the diagnostic accuracy increases when they are used in combination with AFP.

### 5.1. Osteopontin

In 2012, comparative proteomic profiling with the use of mass spectrometry of highly fractionated plasma from patients with cirrhosis and HCC identified osteopontin (OPN) as a new promising biomarker for the early diagnosis of HCC [110]. Additionally, gene expression profiling showed that OPN is one of the leading proteins associated with HCC growth and metastasis [111]. Serum level of OPN is significantly increased even in tumors with a small size, less than 2 cm. OPN expression is dramatically increased in HCC tissues with metastasis correlating with poor OS and recurrence-free survival (RFS) [112].

Osteopontin is a very acidic chemokine-like secreted phosphoglycoprotein found in extracellular matrix (ECM). It is normally expressed in variety of cells and tissues including fibroblasts, osteoblasts, osteocytes, dendritic cells, macrophages, myoblasts, endothelial cells, brain, kidney, and placental cells, where it performs diverse functions [113]. Its normal physiological roles include the involvement in bone mineralization, regulation of immune response, vascular remodeling, wound repair, and control of developmental processes. OPN has been shown to enhance adhesion, migration, invasion, and survival of cells and their attachment to ECM [114].

OPN has been implicated in tumor progression and metastasis through binding of OPN to integrins and CD44 receptors to initiate signaling cascades [115,116]. OPN binds to its receptor integrin αvβ3 to induce autophagy via sustaining FoxO3a stability and to promote cancer stem cell-like phenotype through NF-κB/HIF-1α signaling [117,118]. Analysis of mutational profiles using TCGA revealed that OPN enhances glycolysis in HCC through activating integrin αvβ3/NF-κB/HIF-1α signaling [119]. Additionally, OPN can stimulate epithelial-to-mesenchymal transition (EMT) through Twist-mediated activation of PI3K/Akt signaling [120]. C-C chemokine receptor 1 (CCR1) can be up-regulated by OPN via activation of PI3K/Akt/ HIF-1α signaling [121]. In tumor-associated macrophages (TAMs), OPN promotes programmed death ligand 1 (PD-L1) expression via activation of colony-stimulating factor 1 (CSF1) and its receptor, CSF1R-mediated signaling to facilitate migration and alternative activation of macrophages and to cause immunosuppressive effect [122].

Currently, OPN belongs to phase III biomarkers, which has accuracy and diagnostic value comparable to AFP in the diagnosis of HCC at a cut-off value of 280 ng/mL [123,124]. Despite the diagnostic value of OPN being comparable to that of AFP, sensitivity of OPN can be better than that of AFP in HCC diagnosis. Sensitivity, specificity, and the overall accuracy of the test for OPN can reach up to 100%, 98%, and 96% respectively. The combination of AFP and OPN significantly improves HCC diagnostic performance as compared to AFP alone and elevates both sensitivity and specificity, especially in the early diagnosis of HCC as shown by several systemic meta-analyses [125,126].

Additionally, testing for OPN enables differentiating early-stage HCC from hepatitis B virus-related HCC, hepatitis C virus-related HCC, and liver cirrhosis. The plasma level of OPN in cirrhotic patients has been shown to be higher than that in non-cirrhotic HCV patients. Statistically significant differences in plasma levels of OPN between HCC (401 ± 72 ng/mL) group and non-HCC group are observed when the level of OPN in the control group was 35 ± 6 ng/mL [124]. Combination of OPN with vascular cell adhesion molecule 1 (VSAM-1) has been reported to increase, while OPN and IL-6 to correlate with radiological response after trans-catheter arterial embolization (TAE) [127].

A proteomics approach exploited to analyze the secretory releasing proteome of HBV-associated HCC has showed that among 1365 proteins identified in serum-free conditioned media, levels of AFP, OPN, pregnancy-specific beta-1-glycoprotein-9 (PSG-9) and matrix metalloproteinase-1 (MMP-1), members of transforming growth factor-β (TGF-β)-signaling pathway were the most significantly increased in HCC patients [128]. Mass spectrometry profiling showed an increase in diagnostic performance of the test for HCC when OPN was used in combination with latent-transforming growth factor-β-binding protein-2 (LTBP2). Both OPN and LTBP2 were significantly elevated in HCC patients compared to those with other chronic liver diseases and healthy donors [129]. Meanwhile, LTBP1 alone is also remarkably overexpressed in HCC patients and can show a better diagnostic performance in distinguishing HCC from HVB or cirrhosis as compared to AFP especially in the early staged disease [130].

### 5.2. Glypican-3

Glypican-3 is a heparan sulfate proteoglycan bound to a cell membrane through glycosyl-phosphatidylinositol anchor and first discovered in 2003 to be proposed as a diagnostic and prognostic biomarker for HCC [131]. GPC3 is an oncofetal protein, which can be regulated in the manner similar to that of AFP and can stimulate HCC growth through canonical Wnt/β-catenin-mediated signaling pathway [132,133].

The elevated expression of GPC3 in 283 HCC patients and 445 chronic liver diseases patients as compared to healthy donors has been shown, but there was no difference in GPC levels between HCC and cirrhosis patients [134]. Another study, which enrolled 157 consecutive patients with newly diagnosed HCC to assess diagnostic value of GPC3 as compared to AFP showed that performance of test for glypican-3 in HCC is not satisfactory [135]. Thus, GPC cannot be considered as a promising biomarker for HCC diagnosis and prognosis when used alone. Nevertheless, an introduction of emerging high-throughput imaging techniques and radiomics signature have been proposed as an effective non-invasive and individualized tool to predict GPC3-positive HCC cases correlated with histopathologic grade of the disease [136]. Additionally, several studies showed that GPC3 could be complementary to AFP in increasing diagnostic accuracy of the test for HCC. For example, combination of AFP with GPC3 improved sensitivity of the test for HCC up to 82% or 94%, depending on HCC type [131,137].

Down-regulation of GPC3 with the use of specific siRNAs, miRNAs, or anti-GPC3 antibodies results in a decrease in cancer cell migration, metastasis, and invasion. These data indicate that GPC3 might be a target for anticancer therapy. For example, silencing of GPC3 gene transcription using miR-4510 has been shown to inhibit Ras/Raf/MEK.ERK-mediated signaling and tumor growth [138]. Mostly, miRNAs such as miR-219-5p, miR-485-5P, and miR-194 inhibit HCC progression by targeting GPC3 and inhibition of Wnt/β-catenin signaling [139,140]. In addition to anti-GPC3 antibodies, their conjugation to toxins (immunotoxins) or chimeric antigen receptor T cell (CAR-T) as promising therapeutic strategies are currently being developed for translating into clinical practice [141,142].

### 5.3. Midkine

Midkine (MDK) is a 13-kDA small heparin-binding growth factor detected in the majority of HCC tissues and rarely expressed in surrounding non-tumor tissues [143]. Elevated MDK level has been observed both in the tumor tissue and in the blood samples of HCC patients. Moreover, patients with MDK-elevated HCC have higher amount of circulating tumor cells (CTCs) and significantly higher recurrence rate and shorter RFS [144]. MDK plays an important role in resistance of CTCs to anoikis through activation of PI3K/Akt/NF-kB/TrkB signaling. MDK can be involved in HCC progression and metastasis via ERK/JNK/p38MAPK-mediated signaling promoted by long lncRNA ZFAS1 [145]. The ZFAS1 expression is elevated in HCC but can be suppressed by miR-624.

The sensitivity of MDK for HCC diagnosis is higher than that of AFP even at the early stage of HCC; however, both biomarkers have almost similar specificities, as shown in many studies [146,147]. For example, Zhu et al. showed that AFP and MDK demonstrated specificities of 83.9% and 86.3%, respectively, and the serum level of MDK significantly decreases after curative tumor resection and increases again if tumor relapse occurred [146].

The sensitivity of MDK at a threshold 0.387 ng/mL for HCC diagnosis has been higher than that of AFP at cutoffs of 20, 88.5, and 200 ng/mL, reaching 93.3% in patients with AFP level less than 20 ng/mL [148]. Moreover, in most AFP-negative HCC patients, MDK is overexpressed, and the usage of a combined test for AFP and MDK significantly increases the number of detected HCC cases [149]. A systemic review and meta-analysis study showed that MDK is more accurate in diagnosing HCC, especially in the early-stage and AFP-negative HCC, while both MDK and AFP demonstrated excellent diagnostic performance for hepatitis virus-related HCC [150]. However, meta-analysis data on diagnostic accuracy of MDK is inconsistent due to the limitations in study design and sample sizes [151].

### 5.4. Dickkopf-1

Dickkopf protein (DKK-1) is a secreted glycoprotein and inducer of Spemann’s organizer in Xenopus and can act as an inhibitor of Wnt/β-catenin signaling [152]. Its expression is dysregulated in many malignant tumor types including HCC, multiple myeloma, colorectal adenocarcinoma, etc. [153,154,155]. The elevated expression of both DKK1 and DKK3 in carcinoma tissues of HCC patients as compared to non-carcinoma tissues have been reported [156]. The multi-variant analysis showed significantly longer survival of HCC patients with low DKK1 expression as compared to those with overexpressed DKK1 [157].

Initially, DKK1 was recognized to inhibit Wnt/β-catenin pathway; however, studies show that it has more complex cellular and biological functions [158]. DKK1 has been shown to cause inflammation and to promote cell migration and invasion in HCC through TGF-β1-mediated remodeling of tumor microenvironment. It exerts oncogenic effects in HepG2/C3C cell lines by up-regulating *MYC, CCND1, hTERT,* and *MDM2* oncogenes and down-regulating tumor suppressor genes such as *RB1,* associated with mutation in exon 3 of the *CTNNB1* gene and affect the canonical Wnt/β-catenin signaling pathway [159].

DKK1 is HCC biomarker complementary to AFP in the identification of patients with AFP-negative HCC and distinguishing HCC from non-malignant chronic liver diseases as shown in a large-scale multicenter study [160]. Data obtained using 401 blood samples from 208 HCC patients and 193 liver cirrhosis patients showed that sensitivity and specificity of the tests for AFP were 62% and 90.2%, for PIVKA-II were 51% and 91.2%, for OPN 46.2% and 80.3%, and for DKK1 50% and 80.8%, respectively [161]. However, combined usage of AFP and DKK1 can improve the diagnostic performance for these biomarkers to 78.4% sensitivity and 72.5% specificity.

A meta-analysis of diagnostic accuracy of DKK1 and AFP alone and in combination with each other showed that combined testing for DKK1 and AFP results in the highest accuracy, while DKK-1 alone shows a moderate accuracy in HCC diagnosis [162]. Moreover, combination of three biomarkers including AFP, DKK1, and OPN in a panel showed a better diagnostic performance than AFP alone [163]. Importantly, this combination showed a great improvement in HCC diagnosis at the early stage of the disease. Additionally, the combination of OPN/DKK1 with AFP has been shown to serve as a promising prognostic marker for long-term survival of HCC patients after hepatectomy [164].

### 5.5. Squamous Cell Carcinoma Antigen

Initially, squamous cell carcinoma antigen (SCCA) was discovered as 390 amino acid-containing member of ovalbumin serine protease inhibitor (serpin) family and a tumor marker of squamous cell carcinoma [165]. Afterwards, its two isoforms, SCCA-1 and SCCA-2, encoded by the almost identical tandemly arranged genes, which are not restricted to the squamous epithelium, but can be found in other tissues were reported [166,167]. These serpins may coordinately regulate cysteine and serine proteinase activity in both normal and transformed cells to stimulate cell proliferation and EMT.

Overexpression of SCCA-1 known as SERPINB3 has been found in aggressive HCC with poor prognosis and early tumor recurrence [168]. Mechanisms of tumor growth induced by SERPINB3 include the inhibition of intra-tumor infiltration by natural killer cells, up-regulation of Myc oncogene, and participation in Ras-mediated signaling [169]. Moreover, significant correlation between SCCA-1 and TGF-β expression at both mRNA and protein levels was observed. TGF-β-initiated signaling associated with Wnt target gene expression was also identified as one of the important features of the most aggressive HCCs [170]. Microarray studies showed transcriptional overexpression of SMAD4, the intracellular effector of TGF-β and bone morphogenesis protein (BMP) signaling pathways, in a subset of HCCs [171].

Additionally, SERPINB3 can protect cancer cells from oxidative stress through the up-regulation of HIF-1α transcription and HIF-2α stabilization to favor tumor growth [172,173]. In chronic liver damage, this serpin can lead to HCC promotion through the inhibition of apoptosis, EMT induction, and increase in cell proliferation and invasiveness [174].

Immune complex composed of SCCA and immunoglobulin M (IgM), alone or in combination with AFP, have been proposed as serological HCC biomarkers, which significantly increase sensitivity of HCC diagnosis [175]. Additionally, a significant difference in levels of SCCA-IgM and alpha-L-fucosidase has been observed between HCC and cirrhotic patients suggesting their potential roles as a diagnostic tool to differentiate these two pathologies [176].

Several meta-analyses have been performed to estimate SCCA and SCCA-IgM accuracy for HCC diagnosis. For example, a meta-analysis based on 12 studies showed moderate accuracy of SCCA and SCCA-IgM in HCC diagnosis; however, their combination with AFP is considered as the best diagnostic option [177,178,179]. Simultaneous and combined measurements of AFP, DCP, and SCCA-IgM have been recommended to increase sensitivity, specificity, and diagnostic accuracy of the test for HCC and to make reliable prognosis [180]. Data on HCC conventional and promising biomarkers are summarized in Table 1.

## 6. Screening for Novel HCC Proteomic Biomarker Candidates

Proteomic profiling technologies are currently being extensively utilized to reveal differentially expressed proteins (DEPs) involved in cell signaling, metabolic reprogramming, and ubiquitin-proteasomal degradation during hepatocarcinogenesis. Phosphoproteome and glycoproteome analyses also contribute to candidate biomarker discovery for HCC early diagnosis and prognosis. For example, in a recent study, high performance multiple reaction monitoring mass spectrometry (MRM-MS) was utilized in detecting early-stage HCC within at-risk populations to allow identification of 385 serum HCC biomarker candidates. A multimarker panel consisting of 28 peptides has been created that best differentiated HCC from controls [181]. This multimarker panel showed significantly greater sensitivity (81.1% vs. 26.8%) and lower specificity (84.8% vs. 98.8%) in detecting HCC cases as compared to AFP. 

Another recent global quantitative proteomic analysis of HCC, liver cirrhosis, and non-tumor tissues revealed 33 proteins up-regulated in HCC tissue [182]. Among them, aldo-keto reductase family 1 member B10 (AKR1B10) and cathepsin A (CTSA) in combination with AFP showed the greatest area under the curve (AUC). Additionally, MS-based plasma proteomic atlas containing 53 and 25 molecular biomarkers for HCC and cholangiocarcinoma, respectively, has been constructed to differentiate tumor stage and to assess post-operative prognosis [35]. Six and two HCC biomarkers were abundant in phase II and phase III HCC, respectively, including alpha-2-HS-glycoprotein and apolipoprotein CIII (both ≥0.2%), Ig λ-chain VI region NEWM (≥1.0%), and serum amyloid P component (≥0.3%), which positively correlated with the best OS and RFS.

Further, immunochemistry-based SISPROT proteomic technology allowed accurate and cell-type-specific proteome profiling for the identification of 6660 and 6052 proteins in cancer cells and cancer-associated fibroblasts (CAFs), respectively, in 5 mm^2^ and 12 μm thickness HCC tissues [26]. Among these proteins, cell-type specific ligands and receptors and new potential communications between cancer cells and CAFs have been revealed by bioinformatics analysis. HCC-derived CAFs have been shown to promote cancer cell proliferation and EMT through the overexpression of tissue transglutaminase-2 (TG2) and IL6/IL6R/STAT3 signaling [183].

**Figure 5:**
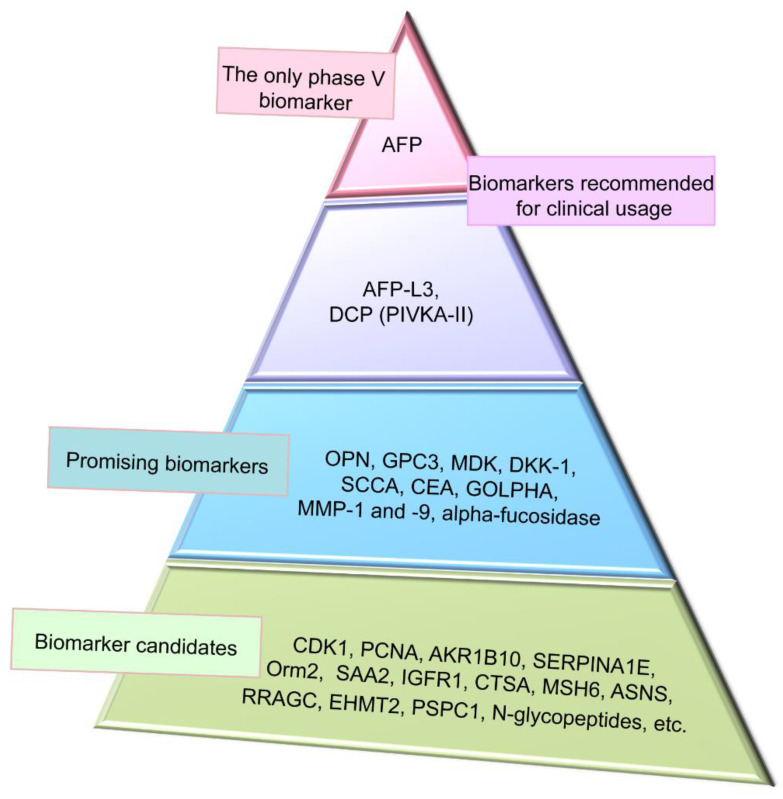
Representation of clinical application of hepatocellular carcinoma (HCC) proteomic biomarkers. Alpha-fetoprotein (AFP) is on the top as the only biomarker, which has passed phase V of clinical trials to be recommended for clinical usage. AFP has low sensitivity from 39 to 64% and specificity from 76 to 91%. Combination of AFP with other biomarkers and imaging techniques significantly improves performance of HCC detection. Gender, age, AFP, AFP-L3, and des-gamma-carboxyprothrombin (GALAD) score is also recommended for clinical usage and has 87% sensitivity and 60% specificity. Promising biomarkers have positions between recommended and candidate biomarkers. Novel HCC biomarker candidates identified by proteomic profiling technologies are needed further verification and validation and are located on the base of the triangle.

Analysis of proteomic data from Clinical Proteomic Tumor Analysis Consortium (CPTAC) and validated in The Cancer Proteome Atlas (TCPA) for 159 patients diagnosed with HBV-related HCC revealed 422 DEPs [184]. Among them were survival-associated proteins including proliferating cell nuclear antigen (PCNA), MutS homolog 6 (MSH6), cyclin-dependent kinase 1 (CDK1), and asparagine synthetase (ASNS) (Figure 5). Additionally, using high pH fractionation and LC-MS/MS analysis, more than 6000 DEPs were identified in IFN-α and INF-λ-stimulated HepG2 cell line under HBV transfection condition [185]. Among these proteins were those involved in interferon signaling, metabolic processes, antiviral response, ubiquitin-proteasomal degradation, and vesicle-mediated transportation. LC-MS/MS approach has been also utilized to reveal diverse ubiquitination patterns of HCC cell lines with different metastatic potential [186].

A recent study performed using proteomic analysis, chromatin immune precipitation assay, and small guide RNA-mediated loss-of-function experiments showed up-regulation of 77 proteins in BIX-01294-treated HCC cells [187]. They include stress-responsive Ras-related GTPase C (RRAGC), which is suppressed by euchromatin histone methyltransferase II (EHMT2) catalyzing dimethylation of histone H3 protein. EHMT2 regulated RRAP expression in ROS generation-dependent manner and has been suggested as a key regulator of stress-responsive genes in HCC.

Among phosphoproteome signatures, receptor tyrosine kinases (RTKs) have attracted a special attention of researchers due to their key regulatory roles in cell proliferation and migration. Integration of proteomics and phosphoproteomics datasets enabled identification of about 176 thousand unique peptide sequences covering about 11,000 protein groups and 32,000 phosphosites [188]. These data were stored and are available in ProteomeXchange repository (http://www.proteomexchange.org, accessed on 28 December 2020). A recent study showed that overexpression of Paraspeckle protein 1 (PSPC1) induces focal adhesion formation and facilitates cell motility via activation of RTK insulin-like growth factor 1 receptor (IGF1R)-mediated signaling [189]. PSPC1 overexpression in tumors has been suggested as a potential biomarker of target therapy with IGF1R inhibitor for improvement of HCC therapy.

Large-scale glycoproteome profiling and quantification of more than 4700 intact *N*-glycopeptides from 20 HCC and 20 paired paracancer samples enabled distinguishing low and high AFP level HCCs. Several sialylated but not core fucosylated tri-antennary glycans have been found to be uniquely increased in HCC with low AFP level, while many core fucosylated bi-antennary or hybrid glycans as well as bisecting glycans were uniquely increased in high AFP level tumors [190]. Differential quantitation analysis revealed that five *N*-glycopeptides at sites N184 and N241 of serum haptoglobin bearing a monofucosylated triantennary glycan were significantly elevated during the progression from non-alcoholic steatohepatitis (NASH) and cirrhosis to HCC. When combined with AFP, the sensitivity for early NASH-related HCC was improved from 59% (AFP alone) to 73% while maintaining a specificity of 70%. These *N*-glycopeptide biomarkers enabled distinguishing 58% of AFP-negative HCC patients from cirrhotic patients [191].

A recent study attempted to identify DEPs in gender-depending hepatocarcinogenesis using Hras12V transgenic mice. In total, 5733 proteins and 1344 DEPs, the common and gender-disparate items, have been identified [192]. Serum amyloid A2 (SAA2), alpha-1-acid-glycoprotein 2 (Orm2), and serine protease inhibitor superfamily member SERPINA1E have been proposed as biomarkers of gender-dependent carcinogenesis in HCC.

## 7. Conclusions

High morbidity and mortality rates of HCC dictate necessity of early and accurate diagnosis of the disease. Over the last decade, the revolution in AI technologies has enormously enhanced HCC diagnosis and prognosis based on the usage of various imaging techniques, especially in combination with HCC molecular biomarkers. However, insufficient specificity and sensitivity of conventional and promising proteomic biomarkers is a major challenge in HCC detection. This is especially important taking into account that HCC is highly heterogeneous and multifactorial tumor. The early diagnosis and prognosis and development of reliable tools for the assessment of therapeutic strategies can increase survival rate for HCC patients. Integrative multiomics (proteomics, genomics, epigenomics, transcriptomics, metabolomics, and peptidomics) approaches are being currently developed for discovery novel biomarkers to improve sensitivity and specificity of the tests for HCC early and accurate diagnosis. Current high-throughput proteomic profiling technologies in combination with AI algorithms (machine learning and deep learning) and predictive models allow exploring novel HCC biomarker candidates to increase sensitivity and specificity of HCC detection and prediction of treatment response for translating into clinical practice. Big data integration provides the best strategy for patients’ health care decision making and gives significant perspectives in HCC precision medicine. However, there has been achieved only limited success in the implementation of AI methods and computational platforms for analysis and interpretation of genomic/proteomic data in cancer detection. Further investigations are needed in this field.

## Figures and Tables

**Table 1:** Conventional and Promising Circulating Proteomic Biomarkers of HCC.

Biomarker	Chemical Nature	Functions	Signaling Pathways	References
AFP and AFL-L3	Embryo-specific and tumor-associated glycoprotein	Dual regulation of cell proliferation and survival	MAPK- an PI3K/Akt/mTOR signaling	[69,70,71,72,73]
DCP	Abnormal prothrombin without carboxylation of γ-carbon atom in Glu residues in γ-carboxyglutamic (Gla) domain	Growth factor activity and DNA synthesis	JAK/STAT3, Raf/MEK1/2/ERK1/2 (MAPK) signaling	[89,90,91]
OPN	Acidic chemokine-like secreted ECM-specific phosphoglycoprotein	Cell adhesion, migration, invasion, and survival, epithelial-to-mesenchymal transition	Integrin αvβ3/NF-κB/HIF-1α and PI3K/Akt/NF-κB and CD44-mediated signaling	[117,118,119,120,121]
GPC3	Heparan sulfate proteoglycan	Cell proliferation and tumor growth	Co-receptor of canonical Wnt/β-catenin signaling; Ras/Raf/MEK/ERK signaling	[138,139,140]
MDK	Small heparin-binding growth factor	HCC progression and metastasis, resistance of CTCs to anoikis	PI3K/Akt/NF-kB/TrkB and ERK/JNK/p38-mediated signaling	[143,144,145]
DKK1	Secreted glycoprotein	TME remodeling, promotion of inflammation, cell migration and invasion	TGF-β1-mediated pathway	[158,159]
SCCA and SCCA-IgM	Member of serine protease inhibitor (serpin) family	Inhibition of apoptosis and intra-tumor infiltration by NK cells; induction of epithelial-to-mesenchymal transition, cell proliferation and invasion	c-Myc and Ras/TGF-β/SMAD4 signaling	[169,170,171]

Notes: AFP, alpha-fetoprotein; AFP-L3, lens culinaris agglutinin (LCA)-reactive L3 glycoform of AFP; DCP, des-gamma-carboxyprothrombin; OPN, osteopontin; GCP3, glypican-3; MDK, midkine; DKK1, dickkopf-1 protein; SCCA, quamous cell carcinoma antigen; IgM, immunoglobulin M.
